# The peers expanding engagement in stimulant harm reduction with contingency management study: a protocol paper

**DOI:** 10.1186/s13722-025-00577-8

**Published:** 2025-06-10

**Authors:** Alexis Cooke, Erin Stack, Linda Peng, Ryan Cook, Bryan Hartzler, Gillian Leichtling, Christi Hildebran, Judith M. Leahy, Kelsey Smith Payne, Lynn E. Kunkel, Kim Hoffman, P. Todd Korthuis

**Affiliations:** 1 Comagine Health, 650 NE Holladay Street, Portland, OR 97232 USA; 2 Department of Medicine, Division of General Internal Medicine, Section of Addiction Medicine, Oregon Health & Science University, Portland, OR USA; 3 Addictions, Drug & Alcohol Institute, University of Washington, Seattle, USA; 4 Health Systems Division, Oregon Health Authority, Portland, OR USA

**Keywords:** Contingency management, Implementation, Stimulant use, Peer support

## Abstract

**Background:**

Contingency management (CM) that is delivered by peer recovery support specialists and incentivizes harm reduction goals among people not seeking treatment for stimulant use has not been tested. The Peers Expanding Engagement in Stimulant Harm Reduction with Contingency Management (PEER-CM) study compares the effectiveness of two peer-facilitated CM interventions: (1) an experimental approach incentivizing achievement of client-identified harm reduction goals and (2) an enhanced standard of care approach incentivizing peer visit attendance.

**Methods:**

Applying a hybrid type 1 effectiveness-implementation framework and stepped-wedge design across 14 community-based peer services sites across Oregon, the PEER-CM study trains peers to conduct CM. All sites implement the standard CM approach of incentivizing peer visit attendance. Every 2 months, two sites are randomly assigned to initiate the experimental CM condition of incentives for achieving client-directed harm reduction activities. Peers monitor progress and manage incentives. In the experimental approach, peers facilitate client progress on goal-related activities (selected from a standardized list of goals) to support the primary study outcome of reducing opioid overdoses and stimulant overamping. The intended study enrollment is approximately 80 clients per site (N = 1,120). Peer specialists participate in skills-focused coaching-to-criterion coaching process to document proficient CM delivery skills. This includes a series of group coaching sessions and an individual assessment with a standardized patient, observed and rated according to core dimensions of the Contingency Management Competence Scale.

**Results:**

The primary study outcome is time until peer-reported fatal or first participant-reported non-fatal overdose or overamp (acute stimulant toxicity). Secondary outcomes include achievement of client-identified harm reduction goals and engagement in substance use disorder treatment. We will also demonstrate the feasibility of our coaching-to-criterion process by documenting peer proficiency in CM skills. Qualitative interviews with peers and their clients will explore the optimal context and implementation strategies for peer-facilitated CM.

**Conclusion:**

PEER-CM is among the first trials to test the effectiveness of peer-facilitated CM for achieving harm reduction goals and reducing overdose in non-treatment-seeking people who use stimulants. The findings will generate evidence for peer-facilitated delivery of CM and application of CM to client-identified harm reduction goals.

*Trial Registration*: This study is registered at ClinicalTrials.gov (NCT 05700994).

**Supplementary Information:**

The online version contains supplementary material available at 10.1186/s13722-025-00577-8.

## Background

Intentional co-use of stimulants and opioids as well as rising fentanyl contamination of methamphetamine (MA) elevates the risks and harms associated with MA use [1]. The age-adjusted rate of drug overdose deaths involving psychostimulants (primarily MA) increased from 0.6% in 2010 to 32.3% in 2021 (0.5 per 100,000) and 2019 (5.0 per 100,000)—a trend that continued and increased during COVID-19 pandemic [13, 16]. Little is known about preventing overdoses among people who use stimulants or engaging people using stimulants in harm reduction and treatment. Strategies to engage people who use stimulants in harm reduction activities (e.g., provision of fentanyl test strips, naloxone, clean syringes, and other supplies) and treatment are needed urgently [18].

Contingency management (CM) uses rewards to incentivize desired behaviors and is the most efficacious strategy for improving retention in treatment for stimulant use disorder (e.g., outpatient treatment, residential rehabilitation, cognitive behavioral therapy) and reducing stimulant use [2, 9]. However, most CM trials and programs are conducted in highly controlled treatment settings [9], and incentivize visit attendance [11, 24, 25] and MA abstinence [9], failing to reach most people who use MA and may not be seeking care or interested in abstinence. Peer recovery support specialists (peers) can reach people not seeking treatment to help them identify harm reduction goals and engage in treatment [30]. Peers are people in recovery, credentialed by the state and trained in harm reduction approaches, who engage people through street and encampment outreach, referrals from emergency departments, hospitals, clinics, syringe service programs, other organizations and self-referrals. There is limited research on the delivery of CM by peers, with one study showing improved outcomes related to HIV milestones [27]. This paper will describe a study protocol combining peer recovery support services (e.g., peer mentoring, resource connection, support groups) with CM for people using stimulants in Oregon.

### PEER-CM study objectives

The Peers Expanding Engagement in Stimulant Harm Reduction with Contingency Management (PEER-CM) study compares the effectiveness of two peer-facilitated CM interventions: (1) an enhanced standard of care incentivizing peer visit attendance, and (2) an experimental approach incentivizing completion of activities related to self-identified goals. The PEER-CM study’s main goal is to test the ability of these CM strategies to reduce overdose through facilitating engagement in harm reduction and treatment services for people using stimulants. The study leverages existing behavioral health funding and a community-based peer organization infrastructure to implement a sustainable peer-delivered CM model for people who use stimulants in rural and urban areas. Study enrollment began in November 2023 and will continue through January 2026.

## Methods

The PEER-CM study uses peer-delivered CM to support non-treatment seeking people who use stimulants to achieve self-identified goals and reduce overdose. Specifically, the PEER-CM study examines the following aims:

1. Test the impact of incentives for achieving self-identified, personal harm reduction goals on the likelihood of overdose among people using stimulants.
2. Determine whether incentives for achieving self-identified, personal harm reduction goals increases engagement with harm reduction and treatment services.

The study was approved by the Oregon Health & Sciences University institutional review board that provides ongoing oversight.

### Oregon’s peer network

Organizations participating in PEER-CM include those in the Peer Recovery Initiated Medical Establishments + Hepatitis B/Hepatitis C/HIV Testing and Linkage to Care (PRIME +) Network. PRIME + connects peers in Oregon with people who are at risk of or are receiving treatment for health issues related to substance use. PRIME + peers provide access to harm reduction supplies, linkage to substance use disorder (SUD) treatment, recovery support, and physical healthcare, support for infectious disease testing and treatment (particularly hepatitis C), access to community resources to meet basic needs, and emotional and crisis support. All PRIME + peers have lived experience of substance use disorder, are certified to provide peer services, and are employed by peer recovery organizations, harm reduction agencies, and health and service organizations to support people who use drugs. The PRIME + Program grew out of a National Institute of Drug Abuse funded pilot program that launched in 2017. This peer program, OR-HOPE (UG3/UH3DA044831), developed a peer harm reduction and engagement intervention to improve access to harm reduction and treatment services to high-needs rural counties with high overdose rates. In 2020, the Oregon Health Authority created the PRIME + Program peer network that scaled up these peer services in 24 of the 36 Oregon counties.

Prior to the launch of PEER-CM, PRIME + expanded to include organizations serving Native American, Black/African American, Latinx/Hispanic and LGBTQIA + communities to address equitable distribution of peer-based harm reduction/treatment engagement-services and contingency management interventions. These newer sites were also included in PEER-CM. The PRIME + Program is funded by Oregon’s Substance Abuse Mental Health Services Administration’s (SAMHSA) State Opioid Response grant (CFDA 93.788 #H79TI085732). The PEER-CM study leverages the PRIME + Program peer network, collaboration, and infrastructure to test the impact of peer-facilitated CM on engaging people who use stimulants in peer services to support achievement of self-identified goals.

### Site selection

PEER-CM utilizes PRIME + sites that demonstrated consistency in data reporting and adequate team supervision. We selected PRIME + sites with prior experience successfully completing federal reporting requirements (e.g. SAMHSA reporting). We also ensured geographic diversity by including sites representing rural Eastern, Coastal, and Southern Oregon. All sites offered PEER-CM participation agreed. In total, nine organizations with 14 sites that provide harm reduction and peer-delivered services were chosen. Of these nine organizations, one supports three PEER-CM sites, and one organization supports 4 PEER-CM sites. Dedicated peers at each site offered services to geographically distinct communities to avoid intervention contamination bias.

### Community-based participatory research approach

The PEER-CM study uses a community-based participatory research (CBPR) approach. Community organizations implementing the intervention are involved in all stages of the research process, including intervention and study design. A community steering committee of administrators and peers from the nine participating community organizations co-developed and guided the implementation of the intervention. The participating organizations held an initial intervention development session in February 2022 to inform the study and convened again in the first quarter of the grant to achieve consensus on the details of CM implementation. The steering committee guided the design of the enhanced standard of care and experimental approaches, considering the needs of their community. This committee also refined the proposed implementation plan during the 6-month start-up phase and the plan will be updated throughout the study with implementation lessons learned and additional feedback. In addition to developing the standardized list of goals and goal activities, the steering committee also convened to determine incentive amounts, number of incentives provided, and the definition of an incentivized peer visit.

### Study design

The PEER-CM study uses a hybrid type 1 effectiveness-implementation framework and stepped-wedge design [17]. Sites were stratified by geographic location (urban/rural) and size (average caseload per month), randomly paired with sites of dissimilar characteristics (e.g., a small, rural site is paired with a larger urban site) and randomized to determine start date of implementing the study’s intervention arm. Randomization was performed by a non-study affiliated statistician; due to the nature of the intervention and study design, masking was not feasible.

All 14 participating sites started PEER-CM enrollment concurrently in the standard of care arm for 3 months. After 3 months, two sites enter the intervention arm at 2-month intervals (i.e., a 2 month “step length”). The PEER-CM study has a 27-month implementation period. All sites implement the standard of care in months 1 through 3. In months 4 through 20, sites enter the intervention arm at randomized 2-month intervals. In months 21–22, sites will complete PEER-CM enrollment, with all sites eventually implementing the intervention arm. In months 23–27, sites continue implementation with enrolled clients. Sites are encouraged to enroll an average of 80 clients, consistently across implementation months (about 4 clients per month). Sites can enroll up to 120 clients throughout the study period.

PEER-CM implementation happens at the site level. When a site starts the intervention arm, all enrolled clients switch to the intervention arm and all newly enrolled clients are exposed only to the intervention arm. If a client enrolls in PEER-CM and remains in the standard of care arm of the study, their study duration will be 6 months total. If a client enrolls in the standard of care arm and their site switches to the intervention arm before 6 months, their study duration may be up to 11 months. Table 1 provides additional details about study duration by site enrollment status. Table 1 depicts sites’ participation in the standard of care and intervention arms throughout the 27-month implementation period. The Standard Protocol Items: Recommendations for Interventional Trials (SPIRIT) flow diagram schedule of enrolment, interventions, and assessment procedures is described in Table 2 [10].

**Table 1:**
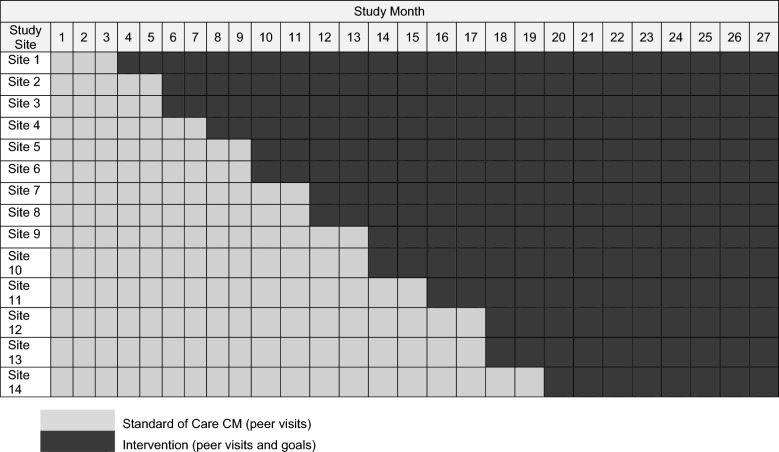
Site study participation

**Table 2:** SPIRIT flow diagram: schedule of enrolment, interventions, and assessment procedures

Timepoint**	Study period					
	Enrolment	Allocation	Post-allocation			Close-out
	−t_1_	0	Intake	Months 1–19	Months 4–27	6-months post enrollment
Enrolment:						
Site enrollment	X					
Informed consent	X					
Stratification of study sites	X					
Site allocation		X				
Interventions:						
Standard of care				X		
Intervention arm					X	
Assessments:						
Intake data • Background information • Locator informatio*n*			X			
GPRA-like survey: • Substance use treatment history, and experiences with overdose or acute events caused by stimulants • Current living situation • Employment, and criminal legal status • Mental health symptoms • HIV and HCV testing • Recovery support connections			X			X
Peer visits (all clients): • Engagement in harm reduction, recovery support, or substance use disorder treatment • Steps clients have made toward meeting their goals • Any recent overdoses and overamps				X	X	
Peer visits (standard): • Client incentives				X	X	
Peer visits (intervention): • Completion of goal-related activities • Incentive provided • Which goal-related activity was completed					X	

### Study arms

 The PEER-CM study uses CM to increase service engagement and retention, and goals completion. Peers are responsible for distributing the incentives and facilitating participation with PEER-CM enrolled clients. In the standard of care arm, peers provide incentives for completing peer visits. In the intervention arm, peers provide incentives for completing peer visits and for completing self-identified goal-related activities.

#### Standard of care arm

The standard of care arm enhances ongoing peer engagement by incentivizing peer visit attendance. Clients in the standard of care arm can receive one $20 gift card every week (i.e., 7 days) for completing up to 15 peer visits (up to $300 over 6 months). The definition of an incentivized “peer visit” was determined with input from the community steering committee including site leadership and peers. A “peer visit” that results in a $20 gift card incentive should be:

- In person (strongly encouraged but not required)
  o Call or video chat is considered a visit, if it’s the only option
  o Text messages are not considered a visit
- One-on-one (peer and client)
- Scheduled or unscheduled
- At least 1 week apart
- Visit duration can vary
- A conversation, check-in that discusses a goal or need (Can be a low-threshold visit)
- Any location as long as other criteria are met

Table 3 lists examples of incentivized and non-incentivized peer visit contacts. Clients can receive swag gifts (e.g. journals, water bottles, notebooks) if they already received a peer-visit gift card during a week, already received 15 peer-visit gift cards over the 6-month period or met with a peer but the visit did not meet all the peer visit expectations defined. Peers do not provide CM incentives for completing goal-related activities in the standard of care arm.

**Table 3:** Peer visit examples

Incentivized contact (1:1)	Non-incentivized contact
Discussion about a need*	Saw client at drop-in center or event, only casual conversation (e.g., Hi, how are you?)
Checking in about a need	Distributed supplies to client, only casual conversation
Support provided to meet need	Run into each other at a recovery meeting and do not have a 1:1 conversation after

^*^Examples of needs: emotional support, transportation/car ride with peer, getting help accessing food, steps toward a goal, have a meal together, go to a meeting together

#### Intervention arm

When a site starts the intervention arm, all clients at a site switch to the intervention arm even if they were enrolled in the standard of care arm. Clients enrolled in the standard of care arm can earn their remaining peer visit incentives and additional incentives for completing self-identified goal-related activities. In the intervention arm, clients can receive up to $599 and receive incentives in two ways: (1) $20 gift card for completing up to 15 peer visits ($300 total) and (2) $30 gift card for completing up to 10 goal-related activities ($300 total). Incentives for peer visits are distributed in the same way as the standard of care arm.

In the intervention arm, peers work with clients to identify personal harm reduction goals and incentivized goal-related activities across six domains: (1) Overdose/Overamping Prevention and Risk Reduction Planning, (2) Substance Use/Recovery Supports, (3) Daily Living, Routines, and Housing, (4) Education, Employment, and Finances, (5) Mental, Physical, and Spiritual Health, and (6) Social, Recreational, Relationships, and Family. This list of goals was developed by the steering committee, see Appendix A, for the list of incentivized goal-related activities. Using the incentivized goal-related activities list, peers and clients identify specific goal-related activities to work towards. Peers then reward clients with CM incentives after verifying that clients complete their goal-related activities. In subsequent peer encounters, peers track goal-related activity completion (maximum of 10 goal-related activities incentivized over 6 months) and record any remaining or revised goals in the PRIME + online database. The revised goals are reviewed and further revised at subsequent visits as needed.

Incentives for completing goal-related activities are provided each time a client verifies completion of an activity related to a self-directed goal. Peers verify completion either through observation or during visits with clients. For example, a client may identify a goal of preventing overdoses (overdose prevention and risk reduction domain). The peer would then reward the client for completing the goal-related activity of overdose prevention training. Another client may have the goal of accessing health care, which would be verified by the peer and client making an appointment together or the peer asking the client the date, time, location of their appointment. If clients have already received their 10 gift cards for goal-related activities completed over the 6-month period or took some steps toward completing the activity but did not fully complete the activity, they can receive swag gifts.

### Client eligibility criteria

PEER-CM inclusion, exclusion, and drop-out criteria are intentionally broad to reach as many clients as possible. Table 4 outlines inclusion and exclusion criteria. Peers are expected to follow up with any enrolled clients for 6 months after enrollment unless a client explicitly says they no longer want to be contacted or involved in PEER-CM.

**Table 4:** PEER-CM eligibility criteria

Inclusion criteria	Exclusion criteria
• Age 18 and older • Any stimulant (methamphetamine, cocaine) use in the past 30 days • Willing to accept peer services • Willing to complete initial and follow-up assessments with peer • Able to communicate in English or Spanish	• Presents any danger or threat of violence to peers

### Client recruitment

Peers recruit clients through their organizations’ current and ongoing outreach activities. This includes street and encampment outreach; referrals from emergency departments, hospitals, clinics, syringe service programs, and other organizations; and self-referrals. During initial conversations with someone who is eligible for PEER-CM, peers use the project overview handouts to describe the PEER-CM program, clarify the goals, and discuss the incentives and swag schedule. Materials are available in English and Spanish. Peers assess client readiness and interest in engaging with the program. While not considered exclusion criteria, peers are trained to assess potential participant fit for PEER-CM. People who may not be a good fit for PEER-CM include individuals who are:

- Ongoing clients who are fully engaged in a treatment or recovery program
- Currently doing well on their current peer services/treatment/recovery plans

In trainings, peers are encouraged to invite people who meet the inclusion criteria to participate and are specifically encouraged to focus on outreach activities and reaching people not already connected to peer services.

## Data collection and measures

### Data collection

All PEER-CM client data are recorded in RecoveryLink, a fully HIPAA-compliant national Electronic Health Record (EHR) platform designed specifically for peer recovery services, developed by Unity Recovery in Philadelphia and used by hundreds of organizations across the United States [3]. Peers enter standardized intake information, modified Government Performance Results Act (GPRA) information, encounter data, peer support activities, and updates on engagement in services. Completion of data entry in RecoveryLink is mandatory for all PEER-CM sites. The study team downloads, deidentifies, cleans, and manages the RecoveryLink data; maintains data quality assurance; and provides monthly site progress reports to organizations, displaying cumulative services results and missing data or discrepancies. Peers enter client data into RecoveryLink upon intake, at check-in visits, and when completing the modified GPRA survey at intake and 6 months after enrollment. All clients consent to data collection in RecoveryLink, as well as use of RecoveryLink data for PEER-CM.

#### Intake 

The intake items in RecoveryLink collect client background information and locator information to aid in maintaining contact with clients. Clients consent to receive services following organizational processes, and consent to participate in PEER-CM is documented during intake.

#### Peer visits

Peers document each incentivized visit in RecoveryLink. This includes recording any updates on engagement in harm reduction, recovery support, or substance use disorder treatment. In the standard of care arm, peers use RecoveryLink to document every time they provide a client an incentive. In the intervention arm, peers document completion of goal-related activities, recording that the incentive was provided, and which goal-related activity was completed from a series of dropdowns. In both arms, peers record steps clients have made toward meeting their goals and document any recent overdoses and overamps (acute stimulant toxicity). Peers complete these items during every incentivized visit with a client and are asked to prioritize these items if time is limited. These items ask:

- Since the last time we talked about issues related to drug use, have you…
  o overdosed? By overdose, I mean if you passed out, turned blue, or stopped breathing from using drugs.
  o felt like you were losing your mind, manic, or psychotic while on methamphetamine or stimulants?
  o felt like you were having a stroke, heart attack, or seizure while on methamphetamine or stimulants?

If yes, peers ask clients to report the date, or a close approximation, of when the overdose(s) and overamp(s) occurred. We supplement self-reported non-fatal overdose with peer reports of fatal client overdoses, as peers tend to follow clients closely in the community and often are among the first to hear about fatal overdoses, however it is possible that some overdoses may not be captured. Peers record nonfatal overdose at peer encounters and record fatal overdose in RecoveryLink as needed.

#### GPRA outcomes survey

The GPRA survey was created by the SAMHSA, which provides funding to PEER-CM and other programs in Oregon and throughout the U.S. The purpose of the survey is to learn how funded services affect clients’ well-being. PEER-CM uses a shorter and adapted version of the GPRA, with questions removed, added, or revised. The GPRA-like survey asks questions about substance use, substance use disorder treatment history, and experiences with overdose or acute events caused by stimulants; current living situation, employment, and criminal legal status; mental health symptoms; HIV and HCV testing; and recovery support connections. To assess changes in outcomes, the GPRA is completed at intake and at 6 months. Peers complete the GPRA intake survey with clients during the first PEER-CM peer services engagement (intake) so that the information reflects the person’s status at or close to the beginning of PEER-CM. To incentivize completion, peers give clients a $25 gift card for completing the 6-month follow-up survey. Clients who switch from the standard of care arm to the intervention arm and engage in PEER-CM for more than 6 months will be asked to complete another GPRA survey at completion of PEER-CM services.

### Data integrity

Participating peer organizations implement and maintain appropriate administrative, physical and technical safeguards to protect client information. Such safeguards comply with federal, state, and local requirements, including 42 CFR Part 2, the Privacy Rule, and the Security Rule. Sites are responsible for maintaining appropriate security regarding all personnel, systems, and administrative processes used to transmit, store and process data. The PEER-CM study team performs regular data quality checks to ensure data entered is complete and accurate. Any recurring issues are addressed one-on-one with peers and their supervisors via email, phone call, or during weekly drop-in hours in which technical assistance is provided.

### Peer training

Peers and peer supervisors receive extensive training through PEER-CM and have opportunities for ongoing training and technical assistance support. Peers and peer supervisors receive continuing education unit (CEU) certificates for all trainings attended. Peers complete three project trainings: (1) Project Overview, (2) CM Skills Training, and (3) PEER-CM Processes, Data Collection Overview, and Data Entry. CM Skills Training include an overview of important CM concepts and coaching-to-criterion of specific CM skills based on six domains of the Contingency Management Competence Scale (CMCS): (1) Informing client of rewards, (2) Outlining future rewards, (3) Providing reward, (4) Assessing interest in rewards, (5) Providing praise, and (6) Linking reward to goals (Nancy M. [24]). CM Skills group trainings include a trainer demonstration, role-plays by peers, and performance-based feedback, adapted from previously validated CM training processes [14, 15].

PEER-CM peers are invited to attend weekly drop-in sessions to discuss any questions. In addition, peers are encouraged to attend regular PRIME + Program events for ongoing support: weekly regional huddles, monthly learning collaborative sessions, weekly peer support for peer support meetings, and annual statewide peer convergence.

## Statistical analysis plan

### Sample size and power

A 46% reduction in the relative likelihood of overdose attributable to the study intervention (a hazard ratio of 0.54) is detectable with > 80% power, assuming a sample size of 14 sites, 3–5 peers per site, and an average of 80 clients per site engaging during the study period (for a total of N = 1,120 clients included in the dataset). A baseline 2-year overdose probability of 25% was assumed, which is likely a conservative estimate given the 6-month rate of 12% observed in pilot data and marked increases in fentanyl use in Oregon since preliminary data was collected [19]. Thus, an 11.5% absolute reduction in the probability of overdose is detectable. This minimally detectable effect size was computed based on the number of sites available to participate, who reported the number of peers employed and average number of clients seen per month. Power calculations were conducted via simulation: “Sites” were randomized to begin the intervention at 2-month intervals, consistent with the research design, and “clients” were assumed to enter the study at uniform random times throughout the study period. Exposure time until overdose, dropout, or study completion was accumulated by each client and allowed to vary based on the intervention activation status of their site. The baseline hazard of overdose was modeled using an exponential distribution such that about 25% of clients would be expected to experience an overdose after 2 years. To account for loss to follow-up, an independent censoring mechanism was built into the simulation. Censoring was assumed to follow a Weibull distribution with decreasing likelihood over time resulting in about 30% loss after 2 years. Modest within-site and peer-within-site correlations in the (log) treatment effect were included, such that the standard deviation of peer-specific treatment effects was about 0.05 (i.e., 95% of peer-specific hazard ratio estimates fell between 0.44 and 0.64). Finally, a two-sided hypothesis test was conducted at a level of significance of 0.05.

### Analysis plan

We will first describe sample demographics, clinical characteristics, and behaviors. Due to the relatively small number of randomized units, we will also carefully examine for imbalance in important overdose prognostic factors (e.g., SUD severity, primary drug of choice) between clients from early and late activating sites. If substantial imbalances in these factors exists, we will use multivariable analyses to adjust and control for covariates. Consistent with best practices for analysis of cluster-randomized stepped-wedge trials, all analyses will account for secular trend in overdose rates (likely, an increasing trend over time as fentanyl becomes more widespread throughout Oregon) and clustering of individuals within sites and peers. Analyses will be conducted with the threshold for statistical significance set at alpha = 0.05.

The primary hypothesis will be tested using a mixed-effects Cox regression model in which time to first overdose (fatal or nonfatal) is the outcome and intervention exposure status (Intervention vs. Standard of Care) is the primary predictor of interest. Exposure status is included as a time-varying covariate to account for clients who spend time in both conditions. The Cox regression model is fit on a calendar time scale to account for secular trends in overdose rates. Random effects for site and peer within site are included to account for clustering. Additional covariates will include stratification factors (site size and geographic location) and other covariates identified as substantially imbalanced between early and late randomized sites as described above. The hazard ratio resulting from this model describes the relative change in the likelihood of overdose resulting from exposure to the intervention. Sensitivity analyses assess the appropriateness of combining (1) likely opioid and acute stimulant toxicity and (2) fatal and nonfatal overdose versus treating them separately in analytic models. As individuals are likely to experience several nonfatal overdoses during the study period, we also plan a secondary analysis allowing for multiple events per participant.

Engagement in harm reduction will be analyzed using a mixed-effects Poisson regression model where the number of self-identified harm reduction goals met is the outcome of interest. Intervention exposure status is the primary predictor of interest, and individuals may have multiple records corresponding to time spent/goals met under each exposure condition. We offset the (log) number of goals set by each participant and include covariates and random effects as described in the primary analysis. We also control for time since enrollment to account for time trends in goal completion, and an additional subject-level random effect is included to account for multiple records per individual. Secondary analyses explore time to meeting first goal, all goals, and specific goals (e.g., time to first completing an overdose prevention training).

Engagement in SUD treatment will be analyzed using a mixed-effects Cox regression model with time to treatment engagement as the outcome. The analytic approach mirrors that of the primary analysis. Secondary analyses will examine time to engagement in medications for opioid use disorder among people with opioid use disorder and other measures of SUD treatment and allowing engagement in multiple treatment episodes/types per client.

### CM fidelity

We will assess CM fidelity using a fidelity criterion validated by previous CM trials involving addiction professionals [14, 15, 23]. CM fidelity will be assessed based on the six domains of the CMCS (as described above in the CM training section). Following CM training sessions, each peer completes a one-on-one role-play with a standardized patient to assess CM skillfulness and fidelity. Trial staff members serving as coaches, rate the six domains of the CMCS according to its Likert scale (1 = Very Poor to 7 = Excellent). Role-plays include feedback and an opportunity for a peer to ‘replay’ specific skills, if necessary. These role plays provide opportunity for the peer to demonstrate adequate CM skillfulness at or above an a priori criterion (a rating of ‘4 = Adequate’ or greater on each of the six CMCS domains). For peer role-plays, we will use descriptive statistics (i.e. mean, range, standard deviation) to analyze the ratings for each domain of the CMCS and CMCS summary score.

## Discussion

The PEER-CM study advances a client-centered, sustainable, scalable, and innovative CM model to increase the impact of harm reduction and treatment engagement services delivered outside of traditional treatment networks and stem the tide of overdose among people who use stimulants. In past studies, clients receiving peer recovery support services demonstrated enhanced substance use treatment engagement, recovery, social determinants of health, and health outcomes, including decreased substance use, increased housing stability, decreased criminal charges, reduced rates of anxiety and tension, and decreased hospital, emergency room, and detoxification center utilization [5, 8, 12, 20]. Historically, harm reduction services and messaging has focused on opioid use and not stimulant use [18, 22]. However, the rise of fentanyl and other synthetic opioids as adulterants in stimulants suggests that efforts to deliver innovative pathways to treatment engagement and harm reduction services to people using stimulants are urgently needed. Results from PEER-CM may inform the use of CM for achieving harm reduction goals, improving treatment engagement, and decreasing overdose in people who use stimulants.

Evidence has consistently supported the use of CM for the treatment of stimulant use disorder [9, 28]. Despite evidence that CM is a cost-effective treatment option, CM adoption in clinical settings remains low. Research indicates that clinicians voice concerns about cost, long-term efficacy, philosophical objections and practical concerns about implementation [26]. Most CM studies are conducted in specialty addiction treatment settings, and dissemination studies have typically focused on CM adoption and implementation by treatment directors, clinical supervisors, and clinicians [6, 7, 11, 15]. However, most people at risk for stimulant-associated overdose and death, are not engaged in the treatment system and many express distrust in traditional structures of healthcare [4, 29]. Peers are able to reach, engage, and retain people actively using drugs in harm reduction services and substance use treatment and are well positioned to elicit and monitor self-identified, personal goals of these populations [5, 8, 21]. However, there remains explicit pushback against peers engaging in CM. A recent U.S Department of Health and Human Services report stated, “…it is not recommended that peer support specialists be permitted to deliver CM” (Contingency Management for the Treatment of Substance Use Disorders: Enhancing Access, Quality, and Program Integrity for an Evidence-Based Intervention, 2023). The PEER-CM study will demonstrate the feasibility and resources required to prepare and maintain peers’ skills to effectively deliver CM interventions while providing important insight on real-world implementation efforts.

## Conclusion

The PEER-CM study is among the first trials to test the effectiveness of peer-delivered CM for achieving harm reduction goals and reducing overdose in non-treatment-seeking people who use stimulants. The findings will generate evidence for peer delivery of CM and application of CM to client-identified harm reduction goals.

## Supplementary Information

Supplementary material 1.

## Data Availability

No datasets were generated or analysed during the current study.

## Abbreviations

PEER-CM: Peers Expanding Engagement in Stimulant Harm Reduction with Contingency Management
CM: Contingency Management
US: United States
MA: Methamphetamine
PRIME +: Peer recovery initiated medical establishments + Hepatitis B/Hepatitis C/HIV testing and linkage to care
SUD: Substance use disorder
SAMHSA: Substance Abuse Mental Health Services Administration
CBPR: Community Based Participatory Research
SPIRIT: Standard Protocol Items: Recommendations for Interventional Trials
EHR: Electronic Health Record
GPRA: Government Performance Results Act
