# Supplementary material for: The peers expanding engagement in stimulant harm reduction with contingency management study: a protocol paper
Source: Addict Sci Clin Pract. 2025 Jun 10;20:48. doi: 10.1186/s13722-025-00577-8 (PMC12150483; doi:10.1186/s13722-025-00577-8)
Supplement: Supplementary file 1 — Supplementary material 1. [file 13722_2025_577_MOESM1_ESM.pdf]

INCENTIVIZED GOAL-RELATED ACTIVITIES

PEER-CM Project: Participant Handout

December 2024

GOALS AND ACTIVITIES ARE ORGANIZED ACROSS THE FOLLOWING 6 DOMAINS:

- Domain 1: Overdose/Overamping Prevention and Risk Reduction Planning
- Domain 2: Substance Use/Recovery Supports
- Domain 3: Daily Living, Routines, and Housing
- Domain 4: Education, Employment, and Finances
- Domain 5: Mental, Physical, and Spiritual Health
- Domain 6: Social, Recreational, Relationships, and Family

Domain 1: Overdose/Overamping Prevention and Risk Reduction Planning

Goal	Incentivized Activity	Activity Completed
**Make plans to prevent overdose / overamping**	▶ Complete **overamping** prevention training and create a written plan<br>• Meet in a place where you can have an in-depth conversation with few interruptions.<br>• Use template provided.<br>• Peer can print plan, send plan by text, or document in RecoveryLink.<br>• Include details about picking up harm reduction supplies, getting naloxone refills, supporting each other, etc. (guidance in template)	☐
	▶ Complete **overdose** prevention training and create a written plan<br>• Meet in a place where you can have an in-depth conversation with few interruptions.<br>• Use template provided.<br>• Peer can print plan, send plan by text, or document in RecoveryLink.<br>• Include details about picking up harm reduction supplies, getting naloxone refills, supporting each other, etc. (guidance in template)	☐
	▶ Do “teach-back” of key counseling handout points (or teach another person) and demonstrate overdose reversal	☐

Goal	Incentivized Activity	Activity Completed
**Update and revise overdose and/or overamping prevention plans**	▶ Review personal plan with peer (including plan for supplies refills) and make updates<br>• Meet in a place where you can have an in-depth conversation with few interruptions.<br>• Occurs at least one month after the overdose/overamping plan was developed.	☐
**Make a plan for substance use or risk reduction**	▶ Create a written substance use/risk reduction plan aside from overdose/overamping<br>• Meet in a place where you can have an in-depth conversation with few interruptions.<br>• Use template provided.<br>• Peer can print plan, send plan by text, or document in RecoveryLink.<br>• Include details about specific activities related to reducing substance use or using more safely; may include plan for supports needed (plan for accessing safer use supplies, changing use to reduce wounds or infectious disease, changing patterns to reduce use, contacting support people, etc)	☐

Domain 2: Substance Use / Recovery Supports

Goal	Incentivized Activity	Activity Completed
**Start treatment of choice**	▶ Schedule intake or appointment with a treatment program or addiction medicine provider	☐
	▶ Attend intake or appointment with a treatment program or addiction medicine provider	☐
	▶ Start medication for substance use like methadone, buprenorphine, naltrexone	☐
**Engage in recovery events**	▶ Attend a mutual support meeting (such as 12-step, Smart Recovery)	☐
	▶ Attend a recovery event or services	☐

Goal	Incentivized Activity	Activity Completed
**Engage in culturally specific services**	▸ Attend a culturally specific event or service	☐
	▸ Attend sweat lodge, talking circle, or drum circle	☐
**Identify a recovery sponsor/elder/support person**	▸ Identify a recovery support person and ask for their phone number	☐
	▸ Meet with recovery support person by phone or in person	☐
**Refer someone else to services**	▸ Bring someone from social circles in or tell someone about services<br>• Can included peer-delivered services, culturally specific services, or other treatment and recovery services	☐

Domain 3: Daily Living, Routines, and Housing

Goal	Incentivized Activity	Activity Completed
**Get driver's license**	▸ Gather information about what is needed to get driver's license. For example:<br>• Go online to locate the steps to getting a driver's license.<br>• Explore if there are any outstanding tickets to be paid.<br>• Go to DMV or online to request the driver's written test workbook.	☐
	▸ Complete requirements to get driver's license. For example:<br>• Pay outstanding ticket(s)<br>• Gather necessary documents (e.g., birth certificate)<br>• Complete the application form<br>• Schedule driving test	☐

Goal	Incentivized Activity	Activity Completed
	▶ Complete driving test	☐
**Get ID card**	▶ Gather information about what is needed to get ID card. For example:<br>• Go online to locate the steps to getting ID card.<br>• Gather required documents for appointment.	☐
	▶ Complete requirements to get ID card. For example:<br>• Gather necessary documents (e.g., birth certificate)<br>• Complete the application form<br>• Schedule appointment	☐
	▶ Go to DMV and complete steps to get ID card	☐
**Improve access to food**	▶ Gather information to sign up for Supplemental Nutrition Assistance Program (SNAP)	☐
	▶ Complete requirements to sign up for SNAP.	☐
	▶ Sign up for SNAP	☐
**Secure housing**	▶ Gather information about programs. For example:<br>• Gather necessary documents (e.g., eligibility paperwork)<br>• Schedule meeting with housing counselor<br>• Meet with housing counselor	☐
	▶ Complete requirements to secure housing. For example:<br>• Gather necessary documents (e.g., birth certificate)<br>• Complete the application form<br>• Schedule appointment	☐

Goal	Incentivized Activity	Activity Completed
	▶ Apply for Public Housing and Housing Choice Vouchers (Section 8) at local Public Housing Agency	☐
	▶ Apply for other housing (e.g., Oxford, low-income)	☐
**Improve legal status**	▶ Attend appointment with probation/parole officer	☐
	▶ Gather information about legal record. For example:<br>• Request information about active warrants	☐
	▶ Complete requirements to improve legal status: For example:<br>• Resolve active warrants<br>• Attend classes (e.g., MRT) and complete other stipulations of probation	☐
	▶ Attend appointment with DHS case worker	☐

Other non-incentivized activities in Domain 3:

- Paying legal or probation/parole monthly fees

Domain 4: Education, Employment, and Finances

Goal	Incentivized Activity	Activity Completed
**Further education**	▶ Gather information about exams, courses, programs	☐
	▶ Complete requirements for this goal. For example:<br>• Complete financial aid forms<br>• Register for classes	☐
**Get specialized training**	▶ Gathering information about certification, trainings, courses, and programs	☐

Goal	Incentivized Activity	Activity Completed
	▶ Complete requirements for this goal. For example:<br>• Complete financial aid forms<br>• Register for programs	☐
**Secure a job**	▶ Gather information about securing a job. For example:<br>• Identify jobs, make lists, circle ads in newspaper<br>• Explore temp agency opportunities<br>• Meet with a job counselor	☐
	▶ Complete requirements for securing a job. For example:<br>• Develop resume<br>• Arrange for references<br>• Gather information to ensure eligibility for employment (e.g., SS card)	☐
	▶ Submit applications at specific places of employment	☐
	▶ Go to job interviews	☐
**Volunteer**	▶ Gather information about volunteer opportunities	☐
	▶ Sign up to volunteer	☐
	▶ Go to volunteer opportunity	☐
**Open a checking / savings account**	▶ Identify bank or credit union	☐
	▶ Open an account	☐

Other non-incentivized activities in Domain 4:

- Go to work regularly
- Miscellaneous (meet with boss, straighten out work schedule, give boss proof of tax status)

Domain 5: Mental, Physical, and Spiritual Health

Goal	Incentivized Activity	Activity Completed
**Prevent or address physical health care concerns**	▶ Enroll in OHP/Medicaid	☐
	▶ Make a doctor (primary care or specialist), dentist, eye appointment	☐
	▶ Attend doctor (primary care or specialist), dentist, eye appointment	☐
	▶ Complete follow-up health care appointment activities. For example:<br>• Fill prescription(s)<br>• Complete recommended testing (e.g., blood draw)<br>• Collect test results	☐
	▶ Get screened for Hepatitis C, HIV, or syphilis	☐
	▶ Start Hepatitis C medication (if applicable)	☐
**Access mental health care and supports**	▶ Schedule intake or appointment with a mental health provider.	☐
	▶ Attend intake or appointment with a mental health provider	☐
	▶ Start medication (if applicable)	☐
	▶ Make plan to help with taking medications consistently, when to contact crisis line, etc.	☐

Goal	Incentivized Activity	Activity Completed
**Build connection to spirituality**	▸ Explore options for building spiritual connections	☐
	▸ Attend events to build spiritual connections	☐

Domain 6: Social, Recreational, Relationships, and Family

Goal	Incentivized Activity	Activity Completed
**Build or strengthen relationships with children and other family members**	▸ Go on outings with (grand)child. For example:<br>• Go to the park<br>• Go to church<br>• Go on a walk	☐
	▸ Visit or assist relative. For example:<br>• Nursing home or prison visit<br>• Take relative to medical appointment	☐
	▸ Write a letter to relative	☐
	▸ Attend social events with specific relatives	☐
**Engage in positive activities**	▸ Make list of leisure, recreational, exercise activities to do. Example of activities may include: go to gym, walk in the park, call friend	☐
	▸ Attend leisure, recreational, or exercise activities	☐
	▸ Complete or work on art/craft	☐
